# Cloning and expression analysis of *Drosophila* extracellular Cu Zn superoxide dismutase

**DOI:** 10.1042/BSR20140133

**Published:** 2014-12-23

**Authors:** Michael J. Blackney, Rebecca Cox, David Shepherd, Joel D. Parker

**Affiliations:** *School of Biological Sciences, University of Southampton, Life Sciences Building 85, Highfield Campus, Southampton SO17 1BJ, U.K.

**Keywords:** alternate RNA splicing, *Drosophila*, extracellular superoxide dismutase, hydrogen peroxide, oxygen free radical, reactive oxygen species, SOD, H_2_O_2_, hydrogen peroxide, NBT, Nitro Blue Tetrazolium, NO, nitric oxide, RNAi, RNA interference, ROS, reactive oxygen species, SOD, superoxide dismutase, *Sod1*, cytoplasmic Cu Zn superoxide dismutase, *Sod2*, mitochondrial Mn superoxide dismutase, *Sod3*, extracellular Cu Zn superoxide dismutase, SOD3v1, SOD3 variant 1, SOD3v2, SOD3 variant 2, TEMED, *N*,*N*,*N*′,*N*′-tetramethylethylenediamine

## Abstract

In the present study, we cloned and sequenced the mRNAs of the *Sod3* [extracellular Cu Zn SOD (superoxide dismutase)] gene in *Drosophila* and identified two mRNA products formed by alternative splicing. These products code for a long and short protein derived from the four transcripts found in global expression studies (Flybase numbers *Dmel\CG9027*, FBgn0033631). Both mRNA process variants contain an extracellular signalling sequence, a region of high homology to the *Sod1* (cytoplasmic Cu Zn SOD) including a conserved AUG start, with the longer form also containing a hydrophobic tail. The two fully processed transcripts are homologous to *Caenorhabditis elegans Sod3* mRNA showing the same processing pattern. Using an established KG p-element+ insertion line (KG06029), we demonstrate that the *Sod3* codes for an active Cu Zn SOD. We found differing expression patterns across sex with higher levels of expression of *Sod3* in females. There is a correlation of *Sod1* and *Sod3* gene expression and activity that can explain why *Sod3* was not seen in earlier studies of *Sod1*. Finally, we found no effect on lifespan with the *Sod3* hypomorph mutation (*Sod3^KG06029^*) but did observe a significant increase in resistance to paraquat and H_2_O_2_ (hydrogen peroxide).

## Abstract 2

Extracellular superoxide dismutase (SOD3) in *Drosophila* is characterized for mRNA splice variants and sex-specific expression. A SOD3 mutant reveals no effect on longevity, enhanced resistance to paraquat and H_2_0_2_, and provided evidence suggesting an interaction with other superoxide dismutases.

## INTRODUCTION

Macromolecular damage caused by the action of superoxide radicals (O_2_^•−^) and other ROS (reactive oxygen species) has long been implicated in the progression of ageing and ill health [1,2]. Although ROS are classically seen to act by causing direct tissue damage, recent work has suggested that ROS may also play a more complex and less direct role in ageing by modifying kinase signalling pathways [3–8].

The SOD (superoxide dismutase) family of enzymes maintain ROS balance by converting one ROS (oxygen free radical, O_2_^•−^) into another [H_2_O_2_ (hydrogen peroxide)], in one of the most catalytically efficient reactions ever measured [9]. H_2_O_2_ is subsequently metabolized by catalase, peroxidases and other scavengers to oxygen and water thereby preventing damage from hydroxyl radicals. However, H_2_O_2_ also functions as a highly diffusible signalling molecule [10–11]. Thus regulation of its turnover may not be as straightforward as simple detoxification. On the other side of the SOD reaction, free radicals can also affect cell signalling by attenuating NO (nitric oxide) signalling and are necessary for long-term potentiation [2,12]. At the same time, NO and superoxide react to produce peroxynitrate which is a highly reactive and dangerous free radical. Thus both the product and reactant of the reaction catalysed by the SOD family of enzymes are capable of causing oxidative damage and affecting signalling pathways. Clearly SODs play a vital role in regulating a wide variety of signalling cascades and therefore need to exert a very constrained regulation to maintain homeostasis.

Like other metazoans, *Drosophila melanogaster* have three compartmentalized SOD isoforms, two of which have been extensively characterized. *Sod1* (cytoplasmic superoxide dismutase) is a Cu (copper) Zn (zinc) binding enzyme and localizes to the cytoplasm [13] and mitochondrial intermembrane space [14]. The *Sod2* (mitochondrial SOD) binds manganese at the catalytic core and is located within the mitochondria [15]. The third, *Sod3* (extracellular SOD) was first identified in *Drosophila* through phylogenetic analysis of SOD DNA sequences after it was cloned from an ant [16]. Prior to this, it was commonly believed that insects lacked the extracellular form [17–19]. The gene has since been confirmed as an extracellular SOD in a parasitic wasp [20] and in *Drosophila* [21]. It was also identified as one of the genes with a significant change in expression in a *Drosophila* model of Alzheimer's disease [22]. In mammals, extracellular SOD is highly expressed in the lung, testis, central nervous system and circulatory system [23,24] and is medically important through its involvement in cardiovascular disease [8,25] and age-related cognitive decline [2,12,26,27].

Mutant analysis of *Sod1* and *Sod2* in *Drosophila* have identified profound effects on lifespan, free radical damage and sensitivity, and age-associated neurological disorders [28,29]. *Sod1*-null mutants show increased susceptibility to oxidative stress by paraquat hypersensitivity, increased DNA damage, infertility as adults and a dramatically reduced lifespan, surviving no more than 3 days [30–32]. Similarly, *Sod2*-null mutants display a reduced longevity, with individuals not surviving past 36 h after eclosion [33]. Furthermore, RNAi (RNA interference) of *Sod2* in *Drosophila* produced the same reduced longevity response [34] suggesting that both SOD1 and SOD2 play key roles in longevity.

Overexpression of these two SODs, however, does not always result in increased lifespan as predicted by the free radical theory of ageing. Initial experiments with *Drosophila* overexpressing native *Sod1* demonstrated no increased resistance to oxidative stress or extended lifespan [35–37]. Overexpression of *Sod2* also failed to increase *Drosophila* lifespan [35,38]. Moreover, *Drosophila* overexpressing *Sod2* and catalase, while being significantly more resistant to oxidative stress, showed a decrease in lifespan of up to 43% [39]. The situation is however not so clear-cut, in other studies overexpression of *Sod1* and/or *Sod2* have been shown to increase *Drosophila* longevity [28,40–42]. Furthermore, mutations in the *Drosophila InR* and *chico* genes, which function in the insulin-like growth factor-like signalling pathway, serve to increase *Drosophila* lifespan in conjunction with raised SOD activity levels [43–45]. While still not fully understood, these contradictory results are now mostly attributed to genetic backgrounds of the *Drosophila* strains used [46,47], therefore the roles are still unknown.

All of the above studies were carried out assuming no other Cu Zn SOD isoforms exist in *Drosophila*. The identification of the third extracellular SOD should attract new interest now that one of its variants has been shown to be up-regulated in a transgenic model of Alzheimer's disease expressing the human amyloid 42 gene [22].

Here we show that *Sod3* is expressed in two isoforms similar to *Caenorhabditis elegans* where membrane-bound and free-floating forms are generating through alternate mRNA splicing [18]. We also found evidence of an uncharacterized interaction between *Sod1* and *Sod3* that can explain why *Sod3* was not observed in earlier studies. Finally, we show that the effect of *Sod3* on lifespan is likely dependent on genetic background and that a mutant *Sod3* gains some protection against both paraquat and H_2_O_2_ exposure. The present study therefore provides an illustration of the importance of balancing the toxicity of product and reactant across membrane compartments for the SODs.

## MATERIALS AND METHODS

### Fly stocks

The laboratory *Oregon R* strain was a kind gift from Dr K. Ubhi (University of California, San Diego, U.S.A.). The *Sod3* hypomorph (*Sod3^KG06029^*) strain (*P{SUPor-P}KG06029*) (stock number 14138) was obtained from the Bloomington Stock Center, Indiana University, Bloomington, U.S.A. This strain was generated by the Gene Disruption Project and contains a single transposon insertion (*P{SUPorP}*) in the 5′ untranslated region of the *Sod3* gene [48]. *Sod3^KG06029^* flies are both viable and fertile and are tested as homozygotes. The *Sod3^KG06029^* line was backcrossed eight times to a control *Bloomington*^yw^ (Bloomington Stock Center) for lifespan experiments. The *Sod1*-mutation lines: *cSod^n108^red/TM3*, *cSod^n108^red/cSod^n108^red*, and *Sod^x39^/TM3* were a kind gift from J.P. Phillips, University of Guelph, Canada.

### *Sod3* cDNA RLM-RACE cloning and sequencing

The *Drosophila Sod3* transcript was cloned from adult poly(A)^+^ RNA (Clonetech, California, US.A.) by RNA Ligase Mediated Rapid Amplification of cDNA Ends (RLM-RACE; Ambion, Warrington, U.K.). Following RNA processing and reverse transcription, the *Sod3* transcript was amplified from the 5′ and 3′ ends of the resulting cDNA by PCR. Nested primers were designed based on the alignment of the GenBank nucleotide sequences for the four predicted *Drosophila Sod3* variants (GenBank accession numbers: NM 165829-variant A; NM 136838-variant B; NM 001038850-variant C; NM 001043071-variant D) with the complete *Drosophila* chromosome 2R genomic sequence (GenBank accession number NT 033778) such that all four should be seen if they were present (3′ outer nested=5′-TTGCCTATCTGATTGGACCCGT-3′, 3′ inner nested=5′-ACGGCTTCCACATTCACGAGAA-3′, 5′ outer nested=5′-CCAATAACACCACACAGGCAATGC-3′, 5′ inner nested=5′-CAACAACTCCCCTGCCAATGAT-3′). PCR conditions were optimized by gradient PCR (94°C, 30 s, denaturation; 55–65°C, 30 s, annealing; 72°C, 30 s, extension) for 35 cycles followed by a 72°C, 7-min final extension. Candidate PCR products to be sequenced were separated by agarose gel electrophoresis with bands excised and purified on Qiagen QIAquick columns. Purified DNA was quantified spectrophotometrically and then subcloned into pGEM®-T Easy Vectors (Promega) for sequencing by Microsynth AG (Switzerland) with the plasmids sequenced in both directions.

### *In silico* analysis of SOD3

Amino acid and DNA sequences were aligned in Bioedit (http://www.mbio.ncsu.edu/bioedit/bioedit.html) with ClustalX [49] (default settings) and checked by eye. Signal-P 3.0 [50] was used to assess for the presence of a signal peptide. A transmembrane index was generated for SOD3v2 (SOD3 variant 2) using the ExPASy Server program, ProtScale [51], and with UMDHMM^TMHP^ [52].

### Transposon excision

The transposable element (*P{SUPor-P}KG06029*) present in the *Sod3^KG06029^* hypomorph was excised using standard techniques where putative excision events were identified by the loss of the white^+^ and yellow phenotype associated with the KG06029 element inserted into the *Bloomington^yw^* background. A number of independent excision lines were established and excision events were first identified through PCR with primers positioned inside the KG element and *Sod3* gene. Because such excisions frequently remove flanking DNA, a subset of clean excisions were identified using a primer set on either side of the KG insertion (*Sod3* del F=5′-CTGAACAATTTGATCGCAGGGC-3′, *Sod3* del R=5′-GGTGGCGCTCTCAATTCTCAAT-3′) giving the expected band size (799 bp). Three of these clean excision lines (*ex136*, *ex141* and *ex158*) were assayed for Cu Zn SOD activity and *Sod3* gene expression (*n*=7 per line) to confirm that CG9027 codes for a *Sod* gene. Statistical analysis of the data was performed using an unpaired *t* test.

### TaqMan® real-time PCR analysis of *Sod* gene expression

Total RNA was isolated from adult *Drosophila* strains using the RNeasy Mini Kit (Qiagen). Using Taqman® Reverse Transcription Reagants (Applied Biosystems), 0.5 μg/μl of each RNA sample was reverse transcribed to cDNA with the resulting cDNA diluted 1:10 in RNase-free H_2_O for use in subsequent real-time PCR reactions. The cDNA sequences for *GAPDH*, *Sod1* and *Sod2* were obtained from GenBank, whereas the cDNA sequences of SOD3v1 (SOD3 variant 1) and SODv2 were determined from the cloning and sequencing of *Sod3* as described in this report. TaqMan® primer and TaqMan-MGB® reporter sequences for each gene (Table 1) were designed from the corresponding cDNA sequences and synthesized by Applied Biosystems. Each real-time PCR assay was performed in triplicate for each gene with each *Drosophila* cDNA sample for a minimum of four biological cDNA replicates (i.e. *n*=4) from each fly strain and sex. *GAPDH* was used as the house-keeping gene to normalize expression. Gene expression was measured in two ways: (1) each gene was quantified as a proportion of *GAPDH* expression for each fly strain; (2) expression was evaluated by comparison with the control strain expression level and displayed as fold up- or fold down-regulated compared with control *Sod* gene expression. Statistical analysis of the data was performed using unpaired *t* tests.

**Table 1:** TaqMan® reporter and primer sequences

Gene	Primer/reporter	Sequence (5′→3′)
*GAPDH*	Forward primer	CGACATGAAGGTGGTCTCCAA
	Reverse primer	ACGATCTCGAAGTTGTCATTGATGA
	Reporter (forward strand)	CTGCCTGGCTCCCC
*SOD1*	Forward primer	CCAAGGGCACGGTTTTCTTC
	Reverse primer	CCTCACCGGAGACCTTCAC
	Reporter (reverse strand)	CCGCTGCTCTCCTGTTC
*SOD2*	Forward primer	GTGGCCCGTAAAATTTCGCAAA
	Reverse primer	GCTTCGGTAGGGTGTGCTT
	Reporter (reverse strand)	CCGCCAGGCTTGCAG
*SOD3v1*	Forward primer	CCAAGAAGACCGGCAATGC
	Reverse primer	GCTGACACGTTGGAAGGGATATTTA
	Reporter (reverse strand)	ACCACAGGCAATGCG
*SOD3v2*	Forward primer	CGCATTGCCTGTGGTGTTATTG
	Reverse primer	GCCACCATCGCGACATG
	Reporter (reverse strand)	CCACATCCGAGTTGATGC

### SOD activity assays

SOD activities were measured according to the principle of Beauchamp and Fridovich [53]. Adult fly samples were prepared as stated elsewhere (approximately 30 flies per sample) for measurement of both total and Cu Zn SOD activity [54]. Application of 2% SDS inactivates Mn SOD allowing for measurement of only Cu Zn SOD activity. Activity assays were performed according to the same protocol; however, the method was modified for a total assay volume of 200 μl which allowed for sample measurement in a 96-well microplate. The reaction was initiated following the addition of 20 μl xanthine oxidase (0.025 units/ml) and measured spectroscopically at 560 nM for 30 min. Each protein sample concentration was tested in quadruplicate and a minimum of four concentrations were tested for each sample such that two inhibited the reaction rate by more than 50% and two inhibited by less than 50%. A minimum of four independent extracts were tested for each genotype. One unit of SOD activity is described as the concentration of protein sample required to inhibit the reaction rate by 50% under our experimental conditions. The protein concentration of each *Drosophila* sample was determined according to the Folin reduction method of Lowry [55]. Statistical analysis of the data was performed using an unpaired *t* test.

### SOD activity gels

In-gel SOD activity was visualized using the NBT (Nitro Blue Tetrazolium) negative staining principle of Beauchamp and Fridovich [53]. Protein samples were assayed by native (non-denaturing) PAGE on a 5% separating gel. A positive control of purified SOD from bovine erythrocytes was also loaded on each gel. Gels were run for 1 h and 45 min at 120 V. Gels were then stained in 10 ml of gel stain solution (12.24 mM NBT, 6.63 M TEMED (*N,N,N*′,*N*′-tetramethylethylenediamine), 0.18 mM riboflavin, 1 M K_2_HPO_4_) in a light-proof box. The box was gently agitated on a rotary shaker at 35 RPM for 20 min. Following staining, gels were illuminated with white light to allow riboflavin within the gel stain to generate O_2_^•−^ in the presence of O_2_ and TEMED. Those areas of the gel lacking SOD became purple-blue due to NBT reduction, whereas regions of the gel where SOD is present remain transparent as SOD scavenges O_2_^•−^. Illumination was maintained until there was maximum contrast between transparent gel and purple-blue areas. A representative example of three trials is shown in Figure 8 inset.

### Survival assay

The backcrossed *Sod3^KG06029^* hypomorph line and the *Bloomington*^yw^ control were kept in an environment control room at 23°C on a 12/12 h light/dark cycle [56]. To collect flies of the same age fresh bottles of flies were set up prior to each experiment. After clearing bottles of adults, pupae were left for 2 days to collect adult flies less than 2 days old. One hundred male flies of each genotype were collected under light CO_2_ anaesthesia and split into vials of ten *Drosophila* each. The vials were maintained in the same conditions as above. Survivors in each vial were scored at least every other day from the day of transfer until population extinction. *Drosophila* were transferred to new food vials once every 7 days without CO_2_ anaesthesia to reduce any effect on lifespan.

### Oxidative stress tests

Three hundred adult male flies up to 2 days of age of each line (*Bloomington^yw^* and back crossed *Sod3^KG06029^*) were collected by light CO_2_ anaesthesia as in the lifespan assay. *Drosophila* were transferred from the stock food bottles into an empty half-pint bottle for each genotype. They were starved in the empty bottles for up to an hour to encourage consumption of the solution when the experiment started.

Five vials of each line were used for each treatment (control, paraquat and H_2_O_2_). One set of five vials each were loaded with two pieces of Whatman filter paper holding 400 μl of 5% (w/v) sucrose solution alone (controls), 400 μl of 5% sucrose solution with 10 mM paraquat or 400 μl of 5% sucrose solution with 15% (v/v) H_2_O_2_. Twenty flies of each genotype were transferred from the empty bottles to these vials under light CO_2_ anaesthesia. Survivors in each vial were scored and any dead removed daily. They were transferred to new vials with fresh filter paper every other day without CO_2_ anaesthesia. The vials were topped up every day with 300 μl 5% sucrose on the days between vial changes and the experiment ran until there was 100% mortality.

### Survival analyses

To analyse the effects of genotype and oxidative stress treatments on lifespan, the Cox Proportional-Hazard Regression Model was used. No more than 2% of the data was censored in each model. 95% confidence intervals (CI) were calculated. In addition, Kaplan–Meier plots were generated and *P* values for Log Rank, Breslow and Tarone-Ware tests were calculated. Cox Regression and Kaplan–Meier models were implemented using the IBM SigmaStat software (SPSS; Systat Software), version 19.0 and again with the R statistics package [57] with R Commander [58] and the Survival plugin [59].

## RESULTS

### *Sod3* expression

**Figure 1:**
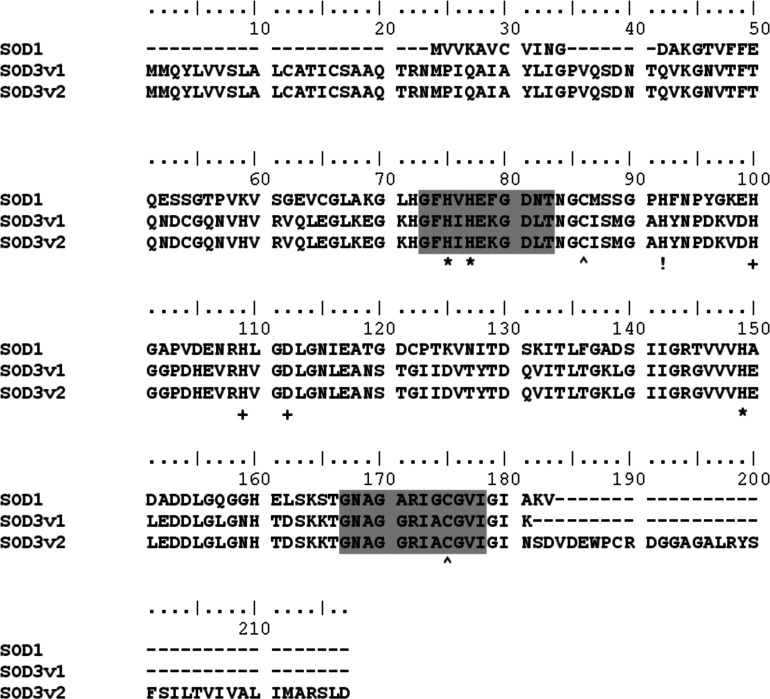
Alignment of *Drosophila* SOD3v1 and SOD3v2 translated peptide sequences with SOD1 Symbol codes: *, Cu-binding sites; ^^^, disulphide bond sites; !, Cu and Zn-binding site; +, Zn-binding sites; shaded regions are the SOD signature sequences.

**Figure 2:**
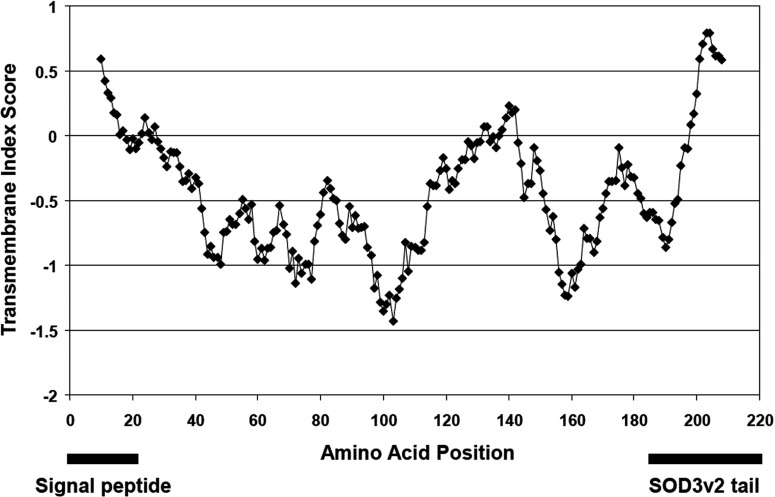
Transmembrane index plot of SODv2 Plot of the 217 amino acids of SOD3v2. Dashed white lines indicate both the hydrophobic N-terminal region predicted to encode the signal peptide, and the SODv2 specific terminal sequence.

Using reverse transcription PCR of adult *Drosophila* poly(A)^+^ RNA with specific *Sod3* nested primers we were able to amplify two mRNA precursors of 859 bp (SOD3v1) and 988 bp (SOD3v2), respectively. Both sequences have been deposited in Genbank (SOD3v1: KM360086, SOD3v2: KM360087). The shorter of the two mRNA species, termed SOD3 variant 1 (SOD3v1) is composed of five exons, whereas the longer species (SOD3v2) is made up of six exons. Translation of the SOD3v1 and SOD3v2 cDNA sequences predicts peptides of 181 amino acids and 19.2 kDa molecular weight, and 217 amino acids and 23.1 kDa molecular weight, respectively. Initial examination of the Flybase *Drosophila* genome lists four EST variants of CG9027 (designated RA, RB, RD and RE in Flybase). Alignments of SOD3v1 and SOD3v2 with these four previously identified transcripts indicates the transcripts from the large-scale projects are most likely partially spliced mRNAs containing introns or fragments of intronic sequence (results not shown). The two variants found here correspond to the short protein product coded by RA and RB (SOD3v1) and the long protein product coded by RD and RE (SOD3v2). The translated SOD3v1 and SOD3v2 sequences aligned with *Drosophila* SOD1 are shown in Figure 1. The two SOD signature sequences, metal binding sites and cysteine cross-linking sites are all conserved. SOD3v1 and SOD3v2 both code for an extracellular signalling sequence (first 23 amino acids) by *in silico* analysis (results not shown). The methionine start site of SOD1 is also conserved in the extracellular transcripts. The longer SOD3v2 includes an additional hydrophobic end (Figure 2) containing a predicted helical peptide transmembrane region with the enzyme predicted to be facing outward (also present in RD and RE).

**Figure 3:**
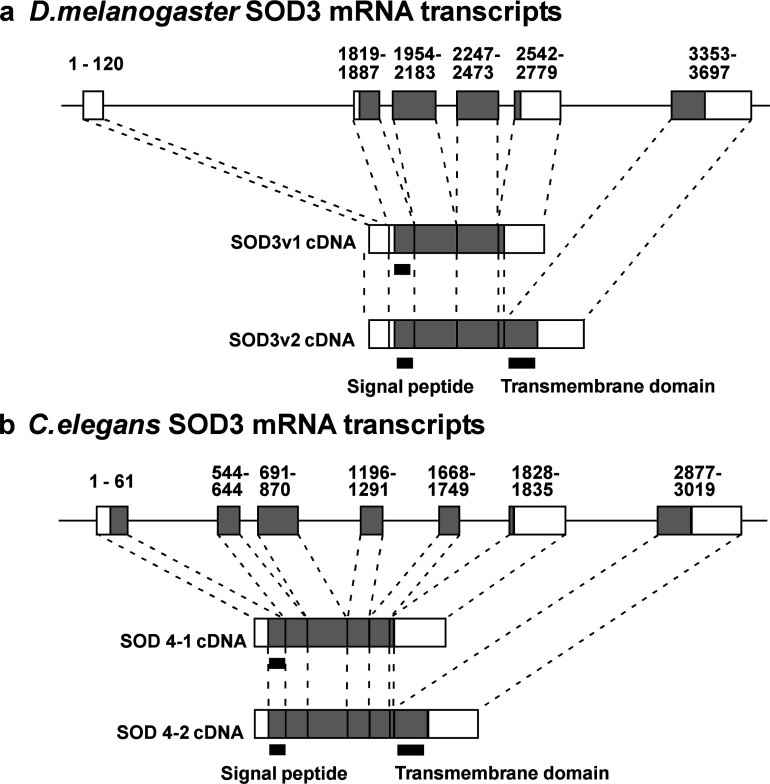
Comparison of alternate splicing patterns of *extracellular* SOD in Drosophila and *C. elegans* The genomic sequence is symbolized as the solid horizontal line. Open boxes represent the non-coding regions and filled boxes are the coding regions of the *Sod3* gene. Number 1-6 and I-VI’ represent exons for each transcript. The numbers below these represent the start and end of the SOD3 nucleotide sequence and include the 5′ and 3′ untranslated regions (UTRs). Diagrams are not drawn to scale. Top (a) is from this study, (b) is a redrawing of Figure 1 from [18] emphasizing the splicing pattern.

Alignment of the two mRNAs with genomic sequences reveals how the two mRNAs are differentially spliced (Figure 3). The pattern is very similar to that seen in extracellular *C. elegans* [18] indicating that SOD3v1 codes for a smaller protein without the hydrophobic transmembrane regions, whereas SOD3v2 codes for the full-length protein.

**Figure 4:**
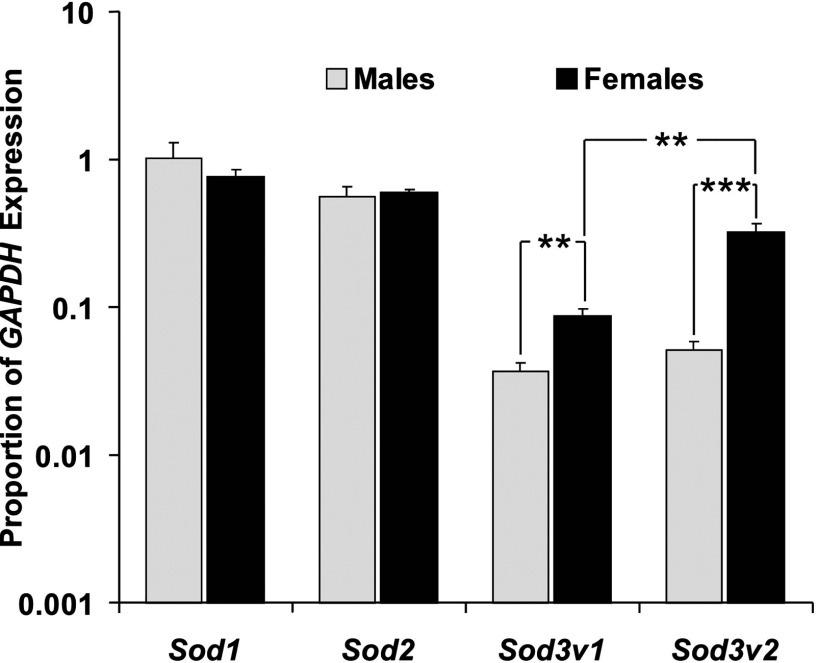
*Sod* gene expression profiles in male and female wild-type flies as a proportion of *GAPDH* expression Five biological replicates of each sex were tested in triplicate for each gene. Error bars are standard error of the mean (S.E.M.). **, *P*<0.01 and ***, *P*<0.001.

Real-time PCR analysis of wild-type expression of SOD3 transcripts revealed extracellular SOD3 is expressed at lower levels than SOD1 and SOD2, with the longer SOD3v2 variant being significantly more highly expressed than the shorter SOD3v1 in both sexes (SOD3v1: d.f.=8, *P*<0.01, SOD3v2: d.f.=8, *P*<0.01) (Figure 4). Females also exhibited a higher level of expression of the longer SOD3v2 transcript compared with males (Figure 4) suggesting a sex-specific increased requirement for the bound form in females (d.f.=8, *P*<0.01).

### Sod3 confers SOD activity

**Figure 5:**
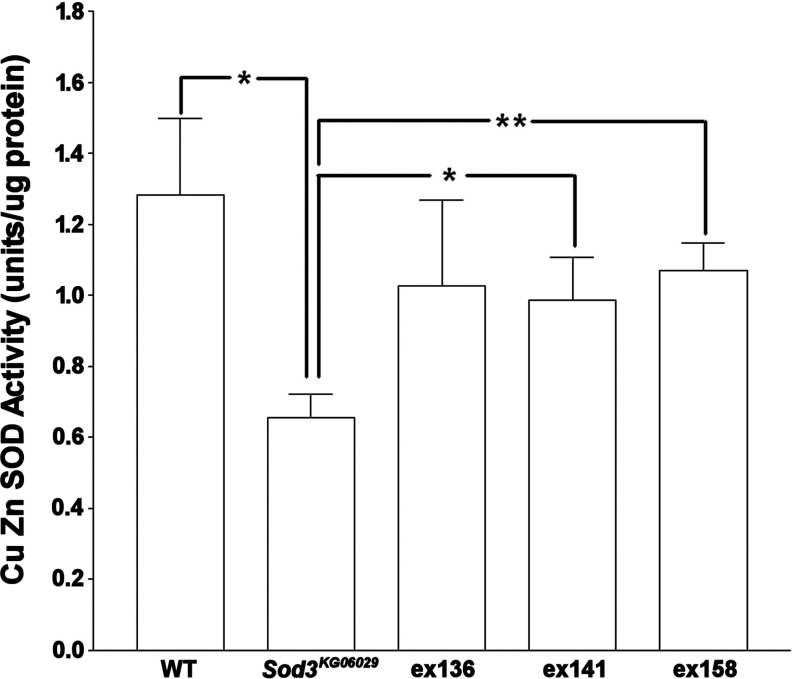
Cu Zn SOD activity assays of transposon excision stocks Excision stocks (*ex136*, *ex141* and *ex158*) were compared with wild-type and *Sod3^KG06029^* flies. Activity was measured in adult mixed sex flies. *, *P*<0.05 and **, *P*<0.005. All samples were tested at *n*=7 and error bars are S.E.M.

The Berkeley Gene Disruption Project fly line *Sod3^KG06029^* has a KG insert just before the start of the SOD3 transcript. This insertion mutant shows lower Cu Zn SOD activity (*n*=5, *P*<0.05, Figure 5) and lower expression than wild-type flies (*n*=5, *P*<0.01 and <0.001) confirming that it has a negative effect on *Sod3* expression/function.

**Figure 6:**
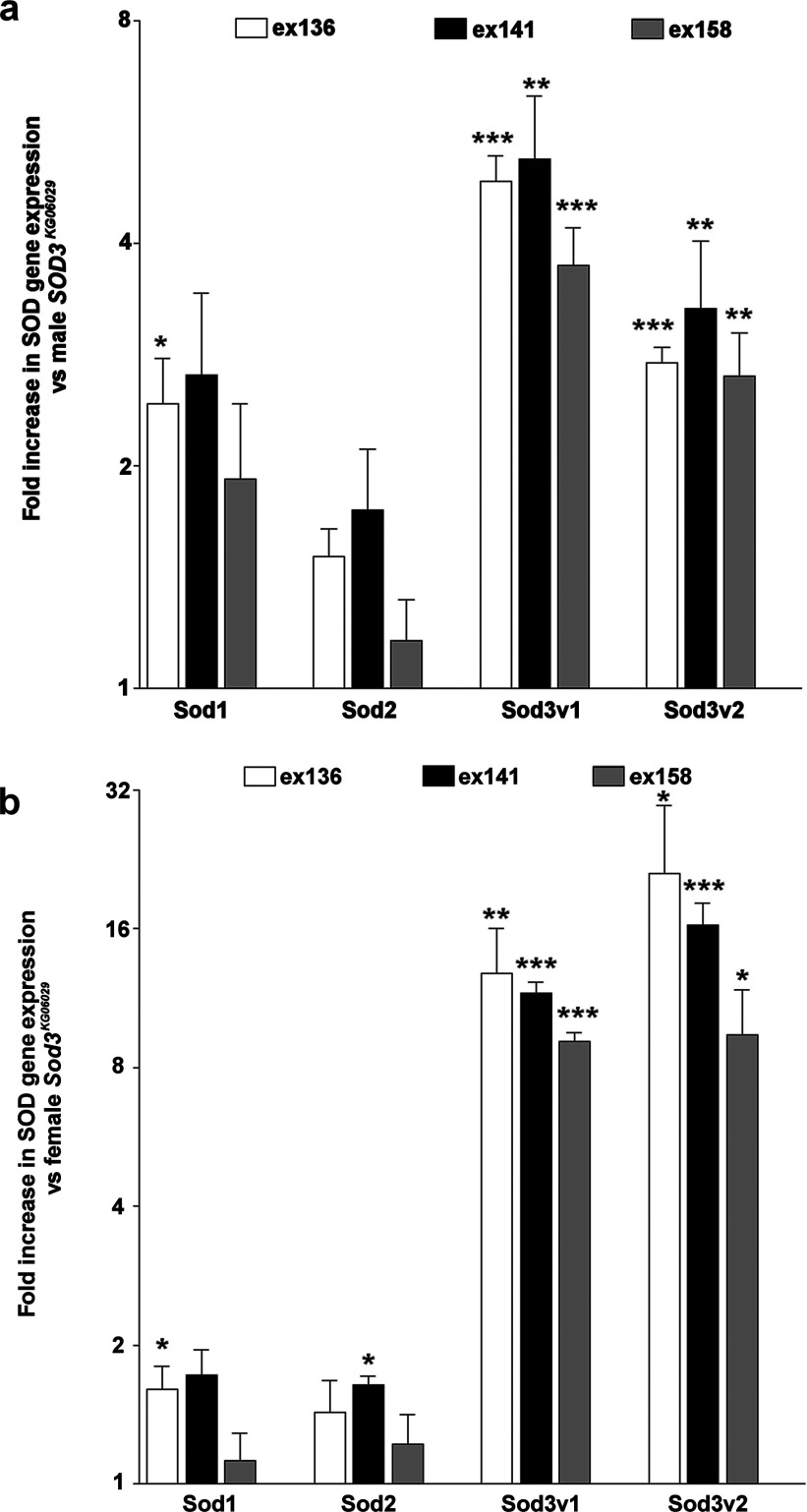
Quantitative real-time PCR of *Sod* gene expression in transposon excision stocks Expression is quantified as fold up-regulation of each *Sod* gene in each excision stock (*ex136*, *ex141* and *ex158*) compared with *Sod3^KG06029^* flies in male (**a**) and female (**b**) flies. Flies were tested at *n*=4 and error bars are S.E.M.

Excision of the KG p-element from *Sod3^KG06029^* (excision verified by PCR, results not shown) restored a wild-type level of Cu Zn SOD enzymatic activity in mixed sex flies as expected (*n*=7, *P*<0.05 and <0.005) for two lines, with the remaining line showing a non-significant shift in the correct direction (Figure 5). We also observed expected increases of SOD3v1 and SOD3v2 expression in all three excision lines (*n*=4, *P*<0.5 to <0.001; depending on line and sex; Figure 6), consistent with restoration of *Sod3* expression after excision. Interestingly, these same excision lines show a tendency for higher mRNA expression for all types of SOD (significant increases in *Sod1* for both sexes in ex136, *n*=4, *P*<0.05, and a significant increase in *Sod2* in ex141 females, *n*=4, *P*<0.05).

### Extracellular SOD expression and activity in historic *Sod1* mutant lines

**Figure 7:**
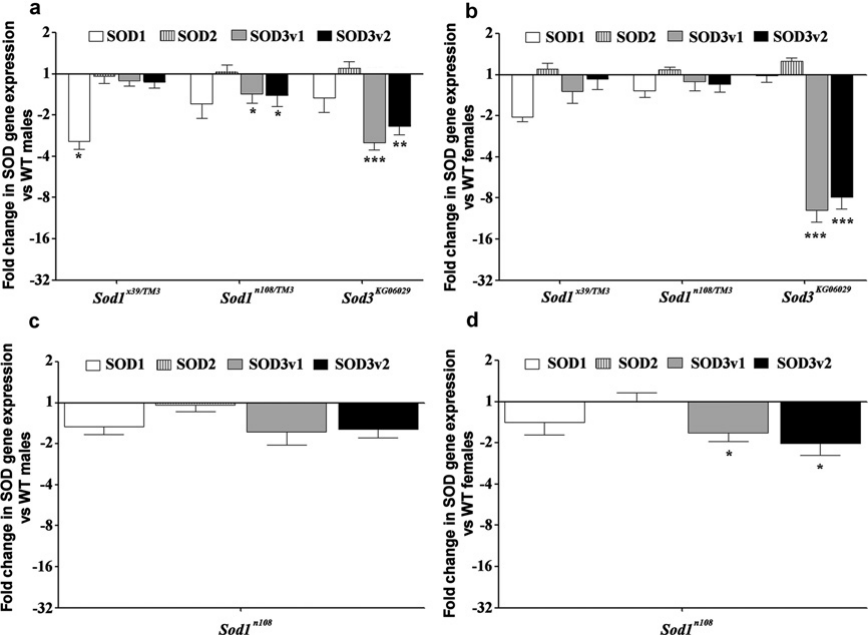
Fold change in *Sod* gene expression in the *Sod* mutant lines **a**=males, **b**=females. *Sod^n108^* homozygotes were run on a different days and thus graphed separately below (**c**=males, **d**=females). All samples are tested at *n*=5 and error bars are S.E.M. *, *P*<0.05; **, *P*<0.01; ***, *P*<0.001.

To understand why SOD3 was not identified in SOD1 studies before the discovery of SOD3, we examined *Sod1* and *Sod3* transcription and resulting SOD activity in the classic *Sod1* mutants, *Sod1^×39^* and *Sod1^n108^*. In both *Sod1* mutant lines, *Sod1* and *Sod3* gene expression tended to be reduced although not significantly in every case. Expression of *Sod1* mRNA is reduced over 2-fold in real-time PCR transcription assays in the heterozygous *Sod1^×39^* deletion line (*n*=5, *P*<0.05 males, *P*<0.001 females; Figures 7A and 7B). Surprisingly the heterozygote of the point substitution mutant (*Sod1^n108^/TM3)* shows significant reduction in *Sod3* mRNA expression of both SOD3 variants in males, and a trend towards less *Sod1* transcription (*n*=5, *P*<0.05; Figure 7A). Changes in the same direction are also observed in females, however in females the SOD1 transcript reduction is significant (*n*=5, *P*<0.05), whereas the two SOD3 variant reductions are not (Figure 7B). Consistent changes in the same direction are observed in the homozygous *Sod^n108^* line with only the levels of the SOD3 variant transcripts in females being significantly reduced (*n*=5, *P*<0.05; Figures 7C and 7D). The homozygous *Sod3^KG06029^* line has much lower expression of the SOD3 variants as expected in both sexes (*n*=5, *P*<0.01 and <0.001; Figures 7A and 7B), but with a suggestion of associated lower expression of SOD1 in males (Figure 7A) and a low but significant up-regulation of the mitochondrial Mn SOD2 in females (*n*=5, *P*<0.01; Figure 7B).

**Figure 8:**
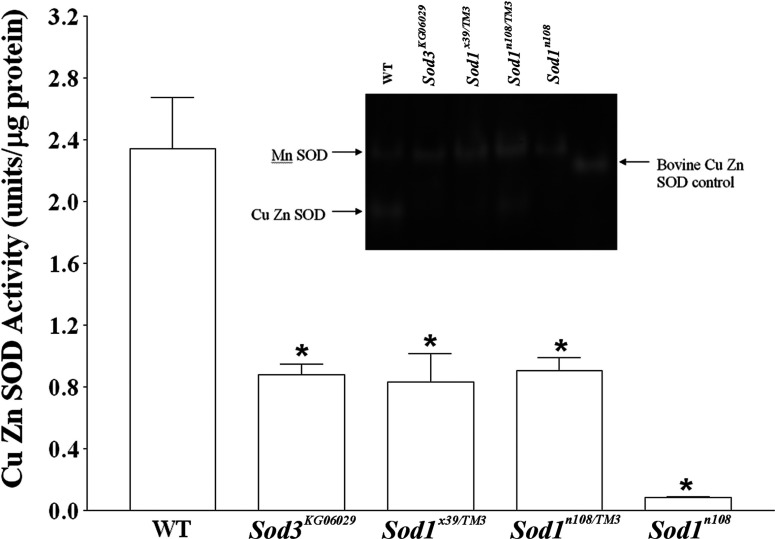
SOD activity measurements in mutant fly lines Cu Zn SOD activities in the same mutant lines were found to be significantly diminished compared with wild-type (all *P*<0.01, d.f.=8, except *Sod1^n108^*, *P*<0.001), however, additionally, a highly significant decrease in activity was also observed between *SOD1^n108/TM3^* and *SOD1^n108^* flies (*P*<0.001, d.f.=8). Wild-type samples were tested at *n*=6 and all other samples were tested at *n*=4. Error bars are S.E.M. Inset, SOD activity gel showing similar results.

Cu Zn SOD enzymatic activity (mitochondrial SOD activity removed) in mixed sex *Drosophila* is reduced by about two-thirds in the SOD1 heterozygous *Sod1^×39^/TM6* and *Sod1^n108^/TM3* mutant lines (d.f.=8, *P*<0.01; Figure 8). This is close to the one-half reduction one would expect for non-functional heterozygotes of a complete knockout (×39) and a recessive point mutation removing almost all activity (n108) if all of the measured Cu Zn SOD activity was attributable to *Sod1*. *Sod1^×39^* homozygotes are not viable so we could not test these. The homozygous *Sod1^n108^* mutants are viable and show a nearly complete loss of all Cu Zn SOD activity (d.f.=8, *P*<0.001 compared with wild-type, and d.f.=8, *P*<0.001 compared with *Sod1^n108^/TM3*; Figure 8). Again, this is consistent with interpreting the assay as only measuring activity from *Sod1*. However, the same magnitude of reduction is also seen in the homozygous *Sod3^KG06029^* line which taken by itself would suggest that SOD3 constitutes about half of the measurable Cu Zn Sod activity. Thus the measured levels of Cu Zn SOD activity in these mutant lines are not consistent with a straightforward additive reduction of activities of the two Cu Zn SODs. This is supported by the results of SOD expression gels with the same *Drosophila* extracts, which show the same proportional reductions of SOD activity in the mutant lines (Figure 8, inset). Thus *Sod1* and *Sod3* expression appears to correlate in the *Sod1^n108^* and *Sod1^×39^* mutant lines.

### No effect of SOD3 mutation on lifespan

**Figure 9:**
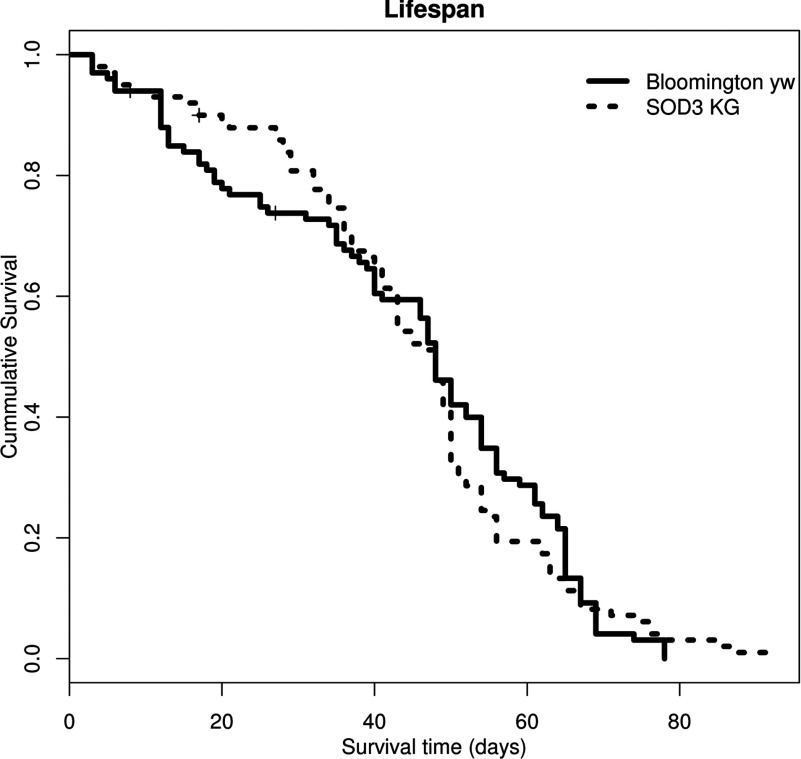
Survival curves (100 flies each) showing no effect on lifespan by the *Sod3^KG06029^* hypomorph No significant difference was detected by Cox regression and Kaplan–Meier analysis. Control is *Bloomington^yw^* and SOD3-KG is *Sod3^KG06029^* backcrossed into *Bloomington^yw^.*

To evaluate whether the *Sod3^KG06029^* mutant has an effect on the average lifespan of *Drosophila*, the cumulative survival of the *Sod3^KG06029^* line was compared against the cumulative survival of *Bloomington^yw^* after eight rounds of backcrossing. No significant difference between the survival patterns of the two genotypes was observed (Figure 9). The Cox Regression Model produced a hazard ratio (HR) of 1.039 (95% CI 0.782–1.381, *P*=0.790). No significant effect was seen with the Log Rank, Breslow and Tarone–Ware tests with *P*=0.781, 0.854 and 0.708, respectively. Mean lifespan for *Bloomington^yw^*=43.849 (95% CI 39.713–47.985) and *Sod3^KG06029^*=44.924 (95% CI 41.262–48.585). The median lifespan was no different between *Bloomington^yw^* (48.000 days, 95% CI 45.249–50.751) and *Sod3^KG06029^* (48.000 days, 95% CI 43.863–52.137). Maximum lifespan at 90% mortality was the same for both lines (67 days).

### Effects of the SOD3 mutation on free radical sensitivity

**Figure 10:**
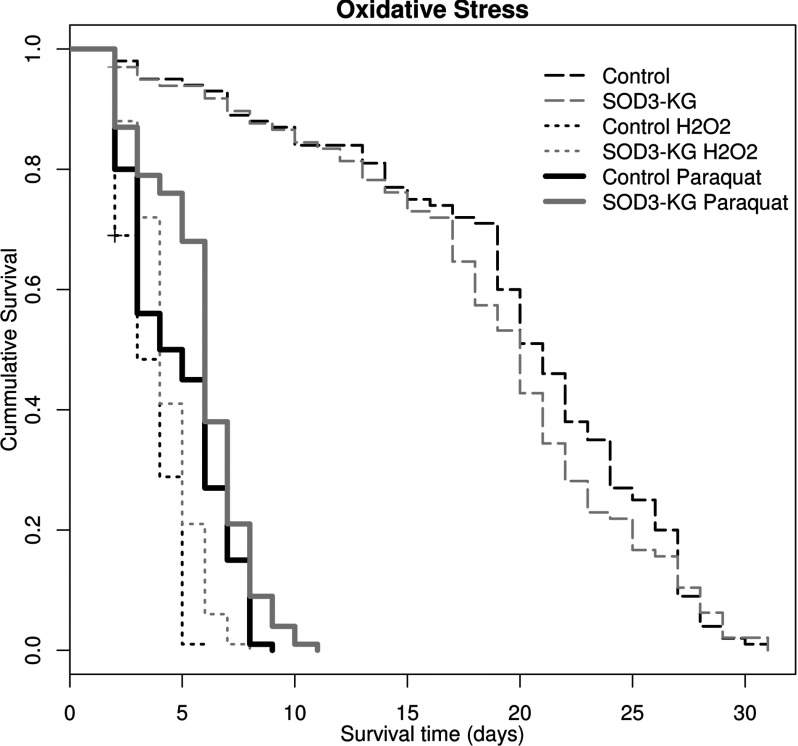
Survival curves showing effects of paraquat and H_2_O_2_ Survival curves (100 flies each) for *Bloomington^yw^* (control) and *Sod3^KG06029^* backcrossed into *Bloomington^yw^* (SOD3-KG) *Drosophila* fed on sucrose with and without H_2_O_2_ or paraquat. No significant difference is observed in the flies fed only sucrose (control, *Sod3^KG06029^* long dashed curves, Cox Regression *P*=0.445). The *Sod3^KG06029^* hypomorph allele significantly protected against both H_2_O_2_ (control H_2_O_2_, *Sod3^KG06029^* small dashed curves, Cox Regression *P*<0.001) and paraquat (control paraquat, *Sod3^KG06029^* solid lines, Cox Regression *P*=0.015).

Paraquat significantly reduced lifespan of both *Bloomington^yw^* (control) and *Sod3^KG06029^* (Cox Regression *P*<0.001 for both lines), while no difference (by Cox Regression) was observed between the two lines fed only on sucrose (Figure 10). Comparison of *Bloomington^yw^* (control) with paraquat and *Sod3^KG06029^* with paraquat revealed that *Sod3^KG06029^* confers significant resistance to paraquat (Cox regression, *P*=0.015, HR 1.420 [95% CI 1.069–1.885], and Log Rank, Brunslow and Tryone–Ware tests all significant at *P*=0.003). Mean lifespan with paraquat for *Bloomington^yw^* was 4.74 days (95% CI 4.307–5.173) and *Sod3^KG06029^* was 5.83 days (95% CI 5.398–6.262). The median lifespans with paraquat have slightly overlapping 95% CI with *Bloomington^yw^*=4.000 days, 95% CI 2.218–5.782, and *Sod3^KG06029^*=6.000 days, 95% CI 5.683–6.317. Maximum lifespan at 90% mortality was the same for both lines with paraquat (8 days).

H_2_O_2_ significantly reduced lifespan of both *Bloomington^yw^* (control) and *Sod3^KG06029^* (Cox regression *P*<0.001 for both lines), while no difference (by Cox Regression) was observed between the two lines fed only on sucrose (the same Kaplan–Meier control plots shown in both; Figure 10). Comparison of *Bloomington^yw^* (control) with H_2_O_2_ and *Sod3^KG06029^* with H_2_O_2_ reveals that *Sod3^KG06029^* also confers significant resistance to H_2_O_2_ (Cox regression, *P*=0.001, HR 1.620 [95% CI 1.207–2.175], and Log Rank, Brunslow and Tryone–Ware tests all significant at *P*<0.001). Mean lifespan with H_2_O_2_ for *Bloomington^yw^* was 3.473 days (95% CI 3.231–3.714) and *Sod3^KG06029^* was 4.290 days (95% CI 4.014–4.566). The median lifespan with H_2_O_2_ was also different between *Bloomington^yw^* (3 days, 95% CI 3.689–4.311) and *Sod3^KG06029^* (4 days, 95% CI 3.689–4.311). Maximum lifespan at 90% mortality for *Bloomington^yw^* was 5 days and for *Sod3^KG06029^* was 6 days.

## DISCUSSION

Through cDNA cloning we have confirmed the presence of two splice variants of the *Sod3* gene, SOD3v1 and SOD3v*2*, in adult *Drosophila*. Although the present study did not determine protein localization, several sequence analysis programmes suggest that the SOD3 enzyme will localize extracellularly [16,21,60]. The two SOD3 splice variants appear homologous to extracellular SOD4 splice variants in *C. elegans* (Figure 3). The extracellular forms of Cu Zn SOD in *C. elegans* (SOD4-1 and SOD4-2) also arise from alternative splicing [18]. SOD4-1 is secreted extracellularly whereas SOD4-2 is secreted and then membrane bound. Both SOD4 peptides contain a putative signal sequence at their N-terminus, while SOD4-2 also contains a hydrophobic membrane-binding domain at its C-terminus. Similarly, a hydrophobicity plot of *Drosophila* SOD3v2 (Figure 2) reveals a hydrophobic terminal sequence at the C-terminus in SOD3v2 (the region missing from SOD3v1). The four mRNA transcripts in genomic studies in Flybase (RA, RB, RD and RE) correspond to the two found here with RA and RB coding for the protein product of SOD3v1 without the hydrophobic tail, and RD and RE coding for the same protein as SOD3v2 with the hydrophobic tail. This, together with the similarity in exon arrangement between *Sod3* in *Drosophila* and *Sod4* in *C. elegans*, suggests that SOD3v1 is a secreted extracellular protein, whereas SOD3v2 is membrane associated. Further evidence suggesting the two forms are functionally different is seen in the results of Favrin [22] who found that the long form RD/RE transcripts are over 10-fold more highly expressed in a *Drosophila* Alzheimer's model, whereas expression of the short form, RA/RB, falls off with ageing similar to the control.

In mammals, two similar functional homologues are generated with a heparin-binding region in place of the invertebrate hydrophobic region and the free form in mammals arises from a proteolytic cleavage [4]. The formation of various combinations of cleaved and non-cleaved extracellular SOD in mammals appears to determine the extracellular binding properties of the SOD tetramer. It is not known whether invertebrate extracellular SOD follows the same pattern by forming such heterotetramers although there is no evidence in the sequence data to rule this out. Given that extracellular SOD in mammals evolved a different mechanism to accomplish the same type of localization capability suggests that the function of extracellular SOD is highly conserved and fundamental. Another conservation from the sequence data is the existence of ATG sites in *Sod3* corresponding with the cytoplasmic *Sod1* gene which could act as an alternative start site for transcription initiation (Figure 1 in site 24). Such conserved sites are present in mice and humans (Figure 1 [18]) opening the possibility that the SOD3 may not always have the extracellular routing sequence and might be cytoplasmic in some instances, but no direct evidence has been found of this to date (Figure 2a in [21]). Whether or not the two Cu Zn SODs have interchangeable localization, the conservation is consistent with Landis and Tower's [60] suggestion that evolution of the two Cu Zn SODs may have involved switching localization. Thus phylogenetic constraint, not function, could account for the conservation of these conserved ATG potential start sites.

Expression of the SOD3 variants was almost 10-fold lower than both SOD1 and SOD2 expression in both sexes of Oregon R wild-type *Drosophila*. The longer membrane binding SOD3v2 is more highly expressed than the shorter free SOD3v1 in females (Figure 4) suggesting a need for more bound SOD3 in female-specific tissue-like ovaries. This is surprising given that a high level of extracellular SOD is found in the male reproductive tract in mammals [24]. Based on mammalian results, one would expect high levels of extracellular SOD in nervous tissue, respiratory tissue and male reproductive tissue. Our results are consistent with those in FlyAtlas showing the highest expression of SOD3 (CG9027 in Flybase) transcripts in nervous tissue, ovaries and hind gut (in descending order) in adult *Drosophila* [61]. This same genome-wide expression database shows SOD3 transcript expressed throughout the life cycle of *Drosophila* with the highest levels detected in larval trachea.

To show that *Sod3* codes for a working SOD we took advantage of the p-element at the beginning of the transcript of CG9027. When this p-element was excised there was an increase in Cu Zn SOD activity to wild-type levels confirming that CG9027 confers SOD activity (Figure 5). There is still SOD3 expression in unexcised *Sod3^KG06029^* so this line is a hypomorph mutant which is not unexpected as the p-element insertion is upstream of the *Sod3* coding sequence.

In addition to transcription levels of the SOD3 variants being elevated in the excision lines there is also elevation in SOD1 and SOD2 expression (Figure 5). This elevation in the other SOD genes in excision suggests that SOD1 and SOD2 may be partially regulated by SOD3 or that modifiers have evolved in the *Sod3^KG06029^* line elevating SOD1 and SOD2 levels in response to less SOD3. However, SOD1 appears significantly up-regulated only after excision of the KG06029 insert (Figure 5) and not before (Figures 7A and 7B) compared with wild-type *Drosophila* controls. In contrast, mitochondrial SOD2 expression is significantly higher before and after excision in females suggesting the effect is due to a modifier. Since all three SODs catalyse the same reaction of O_2_^•−^ to H_2_O_2_, it cannot simply be the expected change in the reactant side of the redox balance leading to up-regulation of SOD1 and SOD2, as reducing the amount of O_2_^•−^ and increasing the amount of H_2_O_2_ with regain of SOD3 would be predicted to lower the overall need for SOD1 and SOD2. Instead, an increase in SOD3 correlates with increasing SOD1 and SOD2 expression levels as a group.

One question to be asked is how *Drosophila* SOD3 went unnoticed despite intense work to understand how the SODs function in *Drosophila* ageing [19,30,36,37,62–65]. To address this we analysed expression and activity of SOD3 in two classic SOD1 mutants: *sod1^x39^* and *sod1^n108^* [19,30,62]. *Sod1^x39^* is lethal as a homozygote, but in the heterozygotes we found an over 2-fold reduction in SOD1 compared with wild-type without a significant effect on SOD2 or SOD3 expression levels (Figures 7A and 7B). The activity level of Cu Zn SOD however is reduced by more than half (Figure 8). In the point mutation mutant *Sod1^n108^* there is a suggestion of lowered *Sod3* mRNA expression in the *Sod1^n108^* heterozygotes with a significant reduction of both SOD3 splice variants in females (Figure 7). This seems paradoxical in that one would not expect a lower demand for Cu Zn SOD in a Cu Zn *Sod* mutant line, unless something else is picking up the function. There is one caveat in that these comparisons are between different *Drosophila* stocks and could result from modifiers.

The picture is more interesting with the enzyme activity data sets. Both the *Sod3^KG06029^* and the heterozygous *Sod1^n108^* reduce Cu ZN SOD activity by a little over half compared with wild-type *Drosophila* just like the *Sod1^×39/TM6^ Drosophila*. The homozygous *Sod1^n108^* however almost entirely abolished all Cu Zn SOD suggesting that both SOD1 and SOD3 are required for Cu Zn SOD activity. Additionally, the reduction in Cu Zn SOD activity observed in *Sod^KG06029^ Drosophila* coupled with restoration of activity in the excision lines demonstrates that we can measure SOD3 activity, and/or SOD3′s effect on SOD1 activity. We suspected that the effects of SOD3 activity was not detected in previous studies because of discarded membranes during tissue processing. However, our data suggest that the SOD1 mutants behaved exactly as if SOD3 did not exist as evidence of SOD3 activity is effectively absent in both *Sod1^x39^* and *Sod1^n108^* lines in assays that clearly measured differences in SOD3 activity in the *Sod3^KG06029^* hypomorph and the *Sod3^KG06029^* excision lines. We did not see similar low expression of *Sod1* in the *Sod3^KG06029^* line but did find a parallel increase in SOD1 and SOD3 with excision. There are several explanations for the correlation of SOD1 and SOD3 expression. First, modifiers may have evolved in the *Sod1* lines that lower the expression of *Sod3*. This explanation does not fit with the observation of increased *Sod1* expression in the KG excision. A second possibility is active regulation and/or interactions of *Sod1* and *Sod3* causing the two to be expressed at similar levels. Either way, whether laboratory adaption of the lines or direct regulation, there is likely some biological reason for the correlated activities of cytoplasmic and extracellular SOD levels seen in Figure 9 that masked insect extracellular SOD.

There is no precedent in the literature to suggest that the Cu Zn SOD genes are linked in any functional way beyond the specialized cellular compartmentalization, so functional interactions of the two Cu Zn SODs must be interpreted with caution in case there are other genetic background effects in play. However, the conserved putative alternative start site for the cytoplasmic and extracellular SODs across the genes in arthropods, mouse and human could place the Cu Zn SODs in the same cellular compartment where they could interact. Indeed, at least one extracellular SOD mutant in mammals interferes with expression and processing of wild-type extracellular SOD in heterozygotes before secretion [66]. Such a mechanism could be working with the *Sod1^n108^* mutation interfering with SOD3, if they are ever in the same location.

Finally, we found no effect on lifespan by the *Sod3^KG06029^* hypomorph. This might seem contradictory to the results of Jung [21] who found great reductions in lifespan with their mutant and RNAi line. The two differences are (1) they assayed at 29°C and 25°C versus the 23°C degree temperature used here; and (2) our mutation is a hypomorph. Either or both of these factors as well as genetic background could account for the results. The more interesting result is the protection observed against paraquat and H_2_O_2_ with the *Sod3^KG06029^* hypomorph. This is logical as paraquat produces free radicals inside the cell, away from SOD3, and once this is converted to H_2_O_2_ by cytoplasmic SOD1 it can diffuse outside the cell. SOD3 then cannot exert any positive influence by scavenging superoxide and can only exacerbate the negative effects of the extra H_2_O_2_. Thus both paraquat and H_2_O_2_ treatments result in extracellular SOD3 being exposed to excessive H_2_O_2_, with both having the same effect. There has been one observation of SOD1 overexpression increasing sensitivity to paraquat that was shown to result from excessive H_2_O_2_ production in *C. elegans* [3]. Excessive H_2_O_2_ deactivates extracellular SOD3 [67], thus a mechanism exists for SOD3 to turn off in the presence of excess H_2_O_2_. If this is protective then less SOD3 means a reduced chance of making too much H_2_O_2_ in high concentrations of extracellular H_2_O_2_. Interestingly, H_2_O_2_ is also implicated in signalling pathways by altering phosphorylation signalling in mammals [68] and in relation to extracellular SOD in *C. elegans* [3]. If normal expression of SOD3 comes with increased sensitivity to ROS then extracellular SOD may function as a regulator of ROS signalling rather than as a detoxification enzyme. Clearly, the regulation, pathways, interactions and function of the conserved SOD gene family in animals may be more complicated than currently thought. Further characterization of *Sod3* will help to complete the story of extracellular SOD in arthropods and resolve the contradictory results concerning SOD and lifespan and lead to further understanding of how this gene family works to maintain ROS signalling across all animals.

## References

[1] Harmon D. (1956). Aging: a theory based on free radical and radiation chemistry. J. Gerontol..

[2] Valko M., Leibfritz D., Moncol J., Cronin M.T.D., Mazur M., Telser J. (2007). Free radicals and antioxidants in normal physiological functions and human disease. Int. J. Biochem. Cell Biol..

[3] Doonan R., McElwee J.J., Matthijssens F., Walker G.A., Houthoofd K., Back P., Matscheski A., Vanfleteren J.R., Gems D. (2008). Against the oxidative damage theory of aging: superoxide dismutases protect against oxidative stress but have little or no effect on life span in *Caenorhabditis elegans*. Genes Dev..

[4] Fattman C.L., Schaefer L.M., Oury T.D. (2003). Extracellular superoxide dismutase in biology and medicine. Free Radic. Biol. Med..

[5] Juarez J.C., Manuia M., Burnett M.E., Betancourt O., Boivin B., Shaw D.E., Tonks N.K., Mazar A.P., Doñate F. (2008). Superoxide dismutase 1 (SOD1) is essential for H_2_O_2_-mediated oxidation and inactivation of phosphatases in growth factor signaling. Proc. Natl. Acad. Sci. U. S. A..

[6] Thiels E., Urban N.N., Gonzalez-Burgos G.R., Kanterewicz B.I., Barrionuevo G., Chu C.T., Oury T.D., Klann E. (2000). Impairment of long-term potentiation and associative memory in mice that overexpress extracellular superoxide dismutase. J. Neurosci..

[7] Chu Y., Alwahdani A., Iida S., Lund D.D., Faraci F.M., Heistad D.D. (2005). Vascular effects of the human extracellular superoxide dismutase R213G variant. Circulation.

[8] Van Deel E.D., Lu Z., Xu X., Zhu G., Hu X., Oury T.D., Bache R.J., Duncker D.J., Chen Y. (2008). Extracellular superoxide dismutase protects the heart against oxidative stress and hypertrophy after myocardial infarction. Free Radic. Biol. Med..

[9] Gray B., Carmichael A.J. (1992). Kinetics of superoxide scavenging by dismutase enzymes and manganese mimics determined by electron spin resonance. Biochem. J..

[10] Martín-Garrido A., Boyano-Adánez M.C., Alique M., Calleros L., Serrano I., Griera M., Rodríguez-Puyol D., Griendling K.K., Rodríguez-Puyol M. (2009). Hydrogen peroxide down-regulates inositol 1,4,5-trisphosphate receptor content through proteasome activation. Free Radic. Biol. Med..

[11] Papaconstantinou J. (2009). Molecular and Cellular Endocrinology Insulin/IGF-1 and ROS signaling pathway cross-talk in aging and longevity determination. Mol. Cell. Endocrinol..

[12] Serrano F., Klann E. (2004). Reactive oxygen species and synaptic plasticity in the aging hippocampus. Ageing Res. Rev..

[13] McCord J.M., Fridovich I. (1969). Superoxide dismutase. An enzymic function for erythrocuprein (hemocuprein). J. Biol. Chem..

[14] Weisiger R.A., Fridovich I. (1973). Superoxide dismutase. Organelle specificity. J. Biol. Chem..

[15] Keele B.B., McCord J.M., Fridovich I. (1970). Superoxide dismutase from *Escherichia coli* B. A new manganese-containing enzyme. J. Biol. Chem..

[16] Parker J.D., Parker K.M., Keller L. (2004). Molecular phylogenetic evidence for an extracellular Cu Zn superoxide dismutase gene in insects. Insect Mol. Biol..

[17] Banks G.K., Robinson A.S., Kwiatowski J., Ayala F.J., Scott M.J., Kriticou D. (1995). A second superoxide dismutase gene in the medfly, *Ceratitis capitata*. Genetics.

[18] Fujii M., Ishii N., Joguchi A., Yasuda K., Ayusawa D. (1998). A novel superoxide dismutase gene encoding membrane-bound and extracellular isoforms by alternative splicing in *Caenorhabditis elegans*. DNA Res..

[19] Phillips J.P., Tainer J.A., Getzoff E.D., Boulianne G.L., Kirby K., Hilliker A.J. (1995). Subunit-destabilizing mutations in *Drosophila* copper/zinc superoxide dismutase: neuropathology and a model of dimer dysequilibrium. Proc. Natl. Acad. Sci. U. S. A..

[20] Colinet D., Cazes D., Belghazi M., Gatti J.L., Poirié M. (2011). Extracellular superoxide dismutase in insects: characterization, function, and interspecific variation in parasitoid wasp venom. J. Biol. Chem..

[21] Jung I., Kim T.Y., Kim-Ha J. (2011). Identification of Drosophila SOD3 and its protective role against phototoxic damage to cells. FEBS Lett..

[22] Favrin G., Bean D.M., Bilsland E., Boyer H., Fischer B.E., Russell S., Crowther D.C., Baylis H.A, Oliver S.G., Giannakou M.E. (2013). Identification of novel modifiers of Aβ toxicity by transcriptomic analysis in the fruitfly. Sci. Rep..

[23] Marklund S.L. (1984). Extracellular superoxide dismutase and other superoxide dismutase isoenzymes in tissues from nine mammalian species. Biochem. J..

[24] Mruk D.D., Silvestrini B., Mo M., Cheng C.Y. (2002). Antioxidant superoxide dismutase–a review: its function, regulation in the testis, and role in male fertility. Contraception.

[25] Foresman E.L., Miller F.J. (2013). Extracellular but not cytosolic superoxide dismutase protects against oxidant-mediated endothelial dysfunction. Redox Biol..

[26] Hu D., Serrano F., Oury T.D., Klann E. (2006). Aging-dependent alterations in synaptic plasticity and memory in mice that overexpress extracellular superoxide dismutase. J. Neurosci..

[27] Levin E.D., Christopher N.C., Crapo J.D. (2005). Memory decline of aging reduced by extracellular superoxide dismutase overexpression. Behav. Genet..

[28] Parkes T.L., Elia A.J., Dickinson D., Hilliker A.J., Phillips J.P., Boulianne G.L. (1998). Extension of Drosophila lifespan by overexpression of human SOD1 in motorneurons. Nat. Genet..

[29] Mockett R.J., Bayne A.C.V, Kwong L.K., Orr W.C., Sohal R.S. (2003). Ectopic expression of catalase in Drosophila mitochondria increases stress resistance but not longevity. Free Radic. Biol. Med..

[30] Phillips J.P., Campbell S.D., Michaud D., Charbonneau M., Hilliker A.J. (1989). Null mutation of copper/zinc superoxide dismutase in *Drosophila* confers hypersensitivity to paraquat and reduced longevity. Proc. Natl. Acad. Sci. U. S. A..

[31] Rogina B., Helfand S.L. (2000). Cu, Zn superoxide dismutase deficiency accelerates the time course of an age-related marker in *Drosophila melanogaster*. Biogerontology.

[32] Woodruff R.C., Phillips J.P., Hilliker A.J. (2004). Increased spontaneous DNA damage in Cu/Zn superoxide dismutase (SOD1) deficient *Drosophila*. Genome.

[33] Duttaroy A., Paul A., Kundu M., Belton A. (2003). A Sod2 null mutation confers severely reduced adult life span in *Drosophila*. Genetics.

[34] Kirby K., Hu J., Hilliker A.J., Phillips J.P. (2002). RNA interference-mediated silencing of Sod2 in *Drosophila* leads to early adult-onset mortality and elevated endogenous oxidative stress. Proc. Natl. Acad. Sci. U. S. A..

[35] Orr W.C., Mockett R.J., Benes J.J., Sohal R.S. (2003). Effects of overexpression of copper-zinc and manganese superoxide dismutases, catalase, and thioredoxin reductase genes on longevity in *Drosophila melanogaster*. J. Biol. Chem..

[36] Orr W.C., Sohal R.S. (1993). Effects of Cu-Zn superoxide dismutase overexpression of life span and resistance to oxidative stress in transgenic *Drosophila melanogaster*. Arch. Biochem. Biophys..

[37] Seto N.O., Hayashi S., Tener G.M. (1990). Overexpression of Cu-Zn superoxide dismutase in *Drosophila* does not affect life-span. Proc. Natl. Acad. Sci. U. S. A..

[38] Mockett R.J., Orr W.C., Rahmandar J.J., Benes J.J., Radyuk S.N., Klichko V.I., Sohal R.S. (1999). Overexpression of Mn-containing superoxide dismutase in transgenic *Drosophila melanogaster*. Arch. Biochem. Biophys..

[39] Bayne A.C., Mockett R.J., Orr W.C., Sohal R.S. (2005). Enhanced catabolism of mitochondrial superoxide/hydrogen peroxide and aging in transgenic *Drosophila*. Biochem. J..

[40] Sun J., Folk D., Bradley T.J., Tower J. (2002). Induced overexpression of mitochondrial Mn-superoxide dismutase extends the life span of adult *Drosophila melanogaster*. Genetics.

[41] Sun J., Molitor J., Tower J. (2004). Effects of simultaneous over-expression of Cu/ZnSOD and MnSOD on *Drosophila melanogaster* life span. Mech. Ageing Dev..

[42] Sun J., Tower J. (1999). FLP recombinase-mediated induction of Cu/Zn-superoxide dismutase transgene expression can extend the life span of adult *Drosophila melanogaster* flies. Mol. Cell. Biol..

[43] Clancy D.J., Gems D., Harshman L.G., Oldham S., Stocker H., Hafen E., Leevers S.J., Partridge L. (2001). Extension of life-span by loss of CHICO, a *Drosophila* insulin receptor substrate protein. Science.

[44] Kabil H., Partridge L., Harshman L.G. (2007). Superoxide dismutase activities in long-lived *Drosophila melanogaster* females: chico 1 genotypes and dietary dilution. Biogerontology.

[45] Tatar M., Kopelman A., Epstein D., Tu M.P., Yin C.M., Garofalo R.S. (2001). A mutant Drosophila insulin receptor homolog that extends life-span and impairs neuroendocrine function. Science.

[46] Spencer C.C., Howell C.E., Wright A.R., Promislow D.E.L. (2003). Testing an ‘aging gene’ in long-lived Drosophila strains: increased longevity depends on sex and genetic background. Aging Cell.

[47] Ziehm M., Piper M.D., Thornton J.M. (2013). Analysing variation in *Drosophila* aging across independent experimental studies: a meta-analysis of survival data. Aging Cell.

[48] Bellen H.J., Levis R.W., Liao G., He Y., Carlson J.W., Tsang G., Evans-Holm M., Hiesinger P.R., Schulze K.L., Rubin G.M. (2004). The BDGP gene disruption project: single transposon insertions associated with 40% of Drosophila genes. Genetics.

[49] Chenna R., Sugawara H., Koike T., Lopez R., Gibson T.J., Higgins D.G., Thompson J.D. (2003). Multiple sequence alignment with the Clustal series of programs. Nucleic Acids Res..

[50] Bendtsen J.D., Nielsen H., von Heijne G., Brunak S. (2004). Improved prediction of signal peptides: SignalP 3.0. J. Mol. Biol..

[51] Gasteiger E., Hoogland C., Gattiker A., Duvaud S., Wilkins M.R., Appel R.D., Bairoch A., Walker J.M. (2005). The Proteomics Protocols Handbook.

[52] Zhou H., Zhou Y. (2003). Predicting the topology of transmembrane helical proteins using mean burial propensity and a hidden-Markov-model-based method. Protein Sci..

[53] Beauchamp C., Fridovich I. (1971). Superoxide dismutase: improved assays and an assay applicable to acrylamide gels. Anal. Biochem..

[54] Mockett R.J., Bayne A.C., Sohal B.H., Sohal R.S. (2002). Biochemical assay of superoxide dismutase activity in *Drosophila*. Methods Enzymol..

[55] Lowry O.H., Rosebrough N.J., Farr A.L., Randall R.J. (1951). Protein measurement with the Folin phenol reagent. J. Biol. Chem..

[56] Iliadi K.G., Iliadi N.N., Boulianne G.L. (2009). Regulation of *Drosophila* life-span: effect of genetic background, sex, mating and social status. Exp. Gerontol..

[57] R Development Core Team (2009). R: A language and environment for statistical computing. R Foundation for Statistical Computing, Vienna.

[58] Fox J. (2005). The R Commander: a basic statistics graphical user interface to R. J. Stat. Softw..

[59] Fox J., Carvalho M.S. (2012). The RcmdrPlugin.survival Package: extending the R Commander Interface to Survival Analysis. J. Stat. Softw..

[60] Landis G.N., Tower J. (2005). Superoxide dismutase evolution and life span regulation. Mech. Ageing Dev..

[61] Chintapalli V.R., Wang J., Dow J.A. (2007). Using FlyAtlas to identify better *Drosophila melanogaster* models of human disease. Nat. Genet..

[62] Campbell S.D., Hilliker A.J., Phillips J.P. (1986). Cytogenetic analysis of the cSOD microregion in *Drosophila melanogaster*. Genetics.

[63] Maria C.S., Revilla E., Ayala A., de la Cruz C.P., Machado A. (1995). Changes in the histidine residues of Cu/Zn superoxide dismutase during aging. FEBS Lett..

[64] Friedman J., Xue D. (2004). To live or die by the sword: the regulation of apoptosis by the proteasome. Dev. cell.

[65] Hari R., Burde V., Arking R. (1998). Immunological confirmation of elevated levels of CuZn superoxide dismutase protein in an artificially selected long-lived strain of *Drosophila melanogaster*. Exp. Gerontol..

[66] Jeon B., Kim B.H., Lee Y.S., Kim S., Yoon J.B., Kim T.Y. (2011). Inactive extracellular superoxide dismutase disrupts secretion and function of active extracellular superoxide dismutase. BMB Rep..

[67] Gottfredsen R.H., Larsen U.G., Enghild J.J., Petersen S.V. (2013). Hydrogen peroxide induce modifications of human extracellular superoxide dismutase that results in enzyme inhibition. Redox Biol..

[68] Goldstein B.J., Mahadev K., Wu X. (2005). Redox paradox: insulin action is facilitated by insulin-stimulated reactive oxygen species with multiple potential signaling targets. Diabetes.

